# Do Subjective Norms Predict the Screening of Cancer Patients’ First-Degree Relatives? A Systematic Review and Meta-Analysis

**DOI:** 10.31557/APJCP.2020.21.6.1521

**Published:** 2020-06

**Authors:** Mojtaba Fattahi Ardakani, Amin Salehi Abargouei, Ahmad Sotoudeh, Somayyeh Esmaeildokht, Vali Bahrevar

**Affiliations:** 1 *Yazd Diabetes Research Center, Shahid Sadoughi University of Medical Sciences, Yazd, Iran. *; 2 *Nutrition Department, School of Public Health, Shahid Sadoughi University of Medical Sciences, Yazd, Iran. *; 3 *Department of Public Health, School of Public Health, Bushehr University of Medical Sciences, Bushehr, Iran. *; 4 *Department Health, Bushehr University of Medical Sciences, Bushehr, Iran. *; 5 *Department of Health Eduacation, Faculty of public health, Shahid Sadoughi University of Medical Sciences, Yazd, Iran. *

**Keywords:** Decision making, relatives, screening, societal norms, cancer

## Abstract

**Background:**

Early detection and preventive measures can reduce the risk of cancer among first degree relatives (FDRs) of cancer patients.Several studies investigated the effect of subjective norm in relation to FDRs’ tendency to conduct preventive behaviors. Therefore, the purpose of this study was to systematically evaluate the effect of subjective norms on cancer patients’ FDRs as well as their willingness for screening.

**Methods:**

PubMed and Scopus were studied to investigate the effect of subjective norms on preventive measures such as breast cancer self-examination, colonoscopy, PSA testing, skin examination, and genetic testing. Odds Ratio (OR), correlation was and confidence intervals were extracted for meta-analysis. After reviewing the studies, only 16 studies met the criteria to be included in this systematic review.

**Results:**

The meta- analysis and OR showed that Physician Recommendation (OR=6.98, 95% CI; 2.55–19.09, P<0.001), Health Care Provider (HCP) (OR=2.79, 95% CI; 1.26-6.16; P=0.011), family and friends (OR=1.82, 95% CI; 1.33–2.50, P <0.001) significantly enhanced the likelihood of referring for screening and preventive measures.

**Conclusions:**

The results of the current study indicated that subjective norms can significantly increase willingness to screening.

## Introduction

Family history is a major risk factor for cancers such as Colorectal (CRC), Breast, Ovarian, Prostate and Melanoma. Cancer risk in men with a First-Degree Relative (FDR) suffering from cancer is higher than men without the family history (Baglietto et al., 2006; Qureshi et al., 2008). The screening and conduct of diagnostic behaviors are carried out with the aim of early detection of disease (Richards et al.,1999) consequently mortality rate will be reduced by preventing complicated diseases; therefore, by an early detection, cancer treatment will be more effective (Khatcheressian et al., 2006; Sherman et al., 2005; Sieverding et al., 2010). Screening may be more critical for individuals who are at a higher risk of the disease (Valeri et al.,2002) but the success depends mainly on how it is conducted. There is a screening method for each of these cancers. In all of them, however, there are external factors such as the pressure of society to encourage them for screening. The intention of screening is one of the predictors of behavior and it can also be predicted by several factors, such as subjective norms (Ajzen, 1985; 1991). In screening promotion studies of most cancers, the conceptual framework includes some combination intentions, self-efficacy, perceived benefit, perceived susceptibility, and subjective norms which are considered the most important determinants of the screening behavior. Subjective norms refer to the personal perception of the social pressures which are imposed to adopt a specific behavior. Similarly, the subjective norm is regarded as a function of the individual’s normative beliefs. Subjective norms refer to the belief that a particular action should be accepted and endorsed by a specific individual or group of people (Ajzen, 2002). 

Although several studies have shown that subjective norms are significantly related to intention, genetic testing or screening in FDRs, they revealed inconsistent results. In most studies, physician recommendation or consultation with a health care provider were significantly related to screening. Furthermore, the analysis of correlation or regression in the Physician Recommendation or friend recommendation showed that they could predict the intention to screening (Boonyasiriwat et al., 2014; Cormier et al., 2003; Madlensky et al., 2003). However, in a cross-sectional study conducted by Hevey, et al., it was reported that the subjective norms did not play any significant role in the screening intention (Hevey et al., 2009). In another study conducted by Glanz et al., (1999) it was reported that among the FDRs recommended by the physician for screening, 23.6 percent had a firm intention of undergoing a genetic test. Despite several published articles, no systematic review and meta-analysis of studies were available regarding the relationship between subjective norms and the intention of the FDRs’ screening. Regarding the vitality of screening in cancer patients’ FDRs, the present study can reduce conflicting results; furthermore, this study could enhance the role of subjective norms in the impact of incentive programs. In the present research, therefore, we have attempted to systematically evaluate and conduct a meta-analysis (Based on PRISMA guidelines) to summarize the data and determine the significance of the relationship between subjective norms and FDR prevention behaviors. 

Also, the study protocol was registered in the international prospective register of systematic reviews (PROSPERO) at CRD42015020240. 

## Materials and Methods

*Search Strategy*

This study was carried out based on the keywords selected from Medical Subject Headings (MeSH) database and non-MeSH terms related to the topic including “Neoplasms”, “Tumor”, “Neoplasia”, “Cancer”, “Benign Neoplasms”, and “Preventive Measures”, “Prevention”, “Early Detection of Cancer”, “Cancer Screening”, “Early Diagnosis of Cancer”, “Screening”, “Participate”, “Intention”, “Willingness”, “Subjective norms”, “Norms”, “Social”, “Subjective Norms”, “Theory of Planned Behavior”, “Health Belief”, “Relatives”, “Siblings”, “Physicians”, and “First-degree relatives”.

Several databases including PubMed, SCOPUS, and ISI web of science up to January 15^th^, 2018, were investigated. The keywords were searched in Google Scholar to ensure that the most relevant publications were investigated. The databases were thoroughly searched without language or date limitations. In the first step, relevant studies were identified with a hierarchical approach based on titles, abstracts and full text features of articles. Then, the reviewers examined the full text feature of all the related articles to check the eligibility of the selected papers. Furthermore, the reference lists of the related articles were checked in order to find other related studies. The first three authors (M.F.A, A.S.A and A.S) followed the procedures individually and any disagreement was resolved through a discussion with M.M.S and S.D as the fourth author.

*Inclusion Criteria*

The following criteria were included in the systematic review and meta-analysis; (i) original, cross-sectional and cohort studies; (ii) investigation of the association between subjective norm and intention to screening cancer patients’ FDRS.

*Exclusion Criteria*

Eligible studies were carefully reviewed for any methodological differences. Research studies with the following specifications were excluded: (i) Studies that investigated the relationship between the FDRs and the community or social support; and (ii) Studies that were conducted on people other than the cancer patients’ FDRs.

*Data Extraction*

The following information was elicited by 2 independent reviewers; the first author’s last name, date of publication, sample size, study design, the population who had genetic testing or screening recommendations, FDRs’ ages and sex, odds ratios (ORs) or correlation coefficients for screening or genetic testing as well as covariates adjusted in the model. In the case of cross-sectional and cohort studies that separately reported ORs for social norms or social recommendations (physician, family, friends, health provider, and patient), the intention and/or /compliance to screening were extracted. 

*Quality Assessment*

 Newcastle-Ottawa Scale adjusted for cross-sectional studies was used to assess the quality of the related articles by a single investigator to examine the quality of the eligible studies (Herzog et al., 2013).

The second investigator also checked the quality assessment results. This scale consisted of three domains: selection (population representativeness, sample size ascertainment of the exposure) with maximum 5 stars, comparability (confounding factors being controlled) with maximum 2 stars and ascertainment of outcome with maximum 3 stars. We decided to assign the highest quality to the top 33.3 percent of the possible scores (7–10); the other studies in the mid 33.3 percent of the possible scores (3–6) were categorized as the medium quality and those in the first 33.3 percent of quality scores (0–2) as low quality. 

*Statistical Analysis*

Coefficients and ORs (and their 95% confidence intervals) were used to calculate Fisher’s Z and Log ORs and their corresponding standard error (SE) was used as an effect size for meta-analysis. Using a random effect model that takes between-study variation into account, the overall effect sizes were calculated. The between-study heterogeneity was investigated using Cochran’s Q test and I-squared. Subgroup analysis was performed based on the person who recommended the cancer screening in the study and the type of cancer to check the possible sources of heterogeneity. In order to find the extent to which inferences might depend on a particular study or a number of publications, sensitivity analysis was used. The publication bias was examined by examining Begg’s Funnel Plots (Egger et al., 1997), checked by formal statistical assessment of Funnel Plot asymmetry by Egger’s Regression Asymmetry Test and Begg’s Adjusted Rank Correlation Test. The meta-regression was employed in order to detect any linear trend between those who were recommended for screening and those who had the intention for screening. The statistical analysis was carried out using STATA version 11.2 (STATA Corp, College Station, TX). P-values of less than 0.05 were considered statistically significant.

## Results

A total of 47,438 articles (36113 in Scopus,8053 in Pubmed and 3,254 in ISI) were initially found, 19, 100 of which remained after omitting the duplicates. By reading the title and abstract, 4,387 articles were excluded and by reading the full text of articles, 270 articles were excluded, which resulted in18 articles for the study (Table1). 

**Figure 1:**
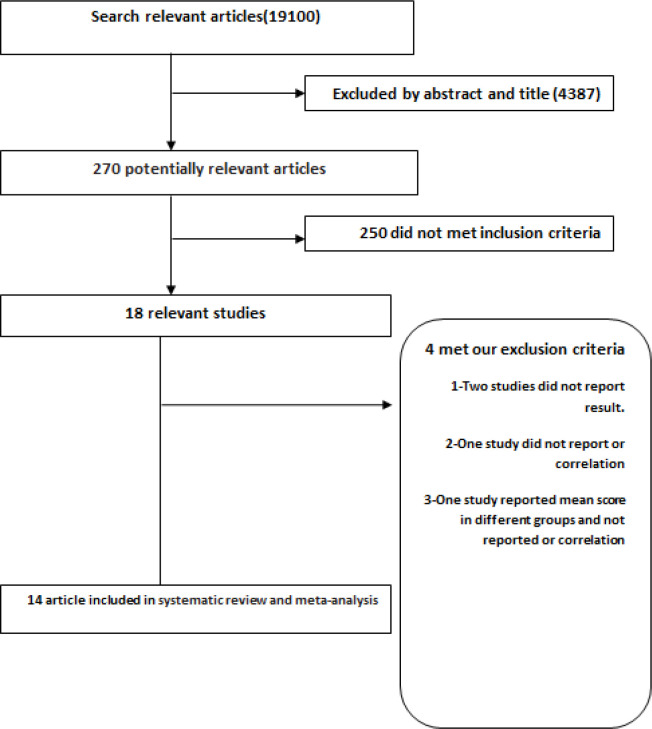
Flow Diagram for Study Selection Process

These 18 observational studies comprising of 2 cohort ( Bennett et al., 2007; Lemon et al., 2006) and 16 cross-sectional studies were included in the systematic review, and 16 studies (out of 16 papers included in systematic review) comprising of 2 cohort studies (Bronner et al., 2013; Ng et al., 2000) and 14 cross-sectional studies were included in the meta-analysis (Figure 1). The correlation between social recommendation and intention or compliance to screening was assessed in 5 studies; odds ratio of screening was investigated based on the social recommendation in 10 studies, and the OR was manually calculated in 2 studies (Glanz et al., 1999; Lemon et al., 2006). Therefore, findings of these studies were included 2 separate studies for the meta-analysis. Studies that did not report ORs or correlation coefficient were included only in the systematic review (Bronner et al., 2013; Gimeno-Garcia et al., 2011; Ng et al., 2000). The selection procedure is summarized in Figure 1.

The studies included in this systematic review are presented in Table 1. Generally, 6,303 participants above 18 years were included in 18 studies and in the systematic review. There were 3 studies on the FDRs with prostate cancer, 3 studies on the FDRs with breast cancer, 9 studies on the FDRs with colorectal cancer, and 3 studies on the FDRs with melanoma; however, 4 studies were not included in meta-analysis (Bronner et al.,2013; Gimeno-Garcia et al., 2011; Ng et al., 2000). In Gimeno-Garcia study, among FDRs, individuals who used to visit their family doctor had a tendency toward better participation but this association was not statistically significant, due to the small sample size (Gimeno-Garcia et al., 2011). One study introduced the subjective norm as a determinant of compliance with the BSE (Breast self- examination), yet the results including the factors in BSE compliance were not reported (Ng et al., 2000). In a study conducted by Hevey, the subjective norm could not explain intention to screening, neither did it provide data for meta-analysis (Hevey et al., 2009). In Bronner et al. study, the social pressure as a motivational factor expectation and view of friends, colleagues and neighbors did not influence the FDRs’ decision to undergo colonoscopy screening (Bronner et al., 2013). Therefore, four aforementioned studies were not included in the meta-analysis. 

*Quality Assessment of Included Studies*

 The result of quality assessment based on the modified Newcastle-Ottawa scale is reported in Table 2. In brief, all of studies included studies achieved relatively high scores.

*Results from Meta-Analysis*

Physician recommendation and compliance with screening:

Out of 16 studies, nine had data regarding the PR in relation to screening and 8 found significant results. Meta-analysis revealed that the PR significantly refers to screening (OR=6.98, 95% CI; 2.55–19.09; P<0.001).

**Figure 2.**
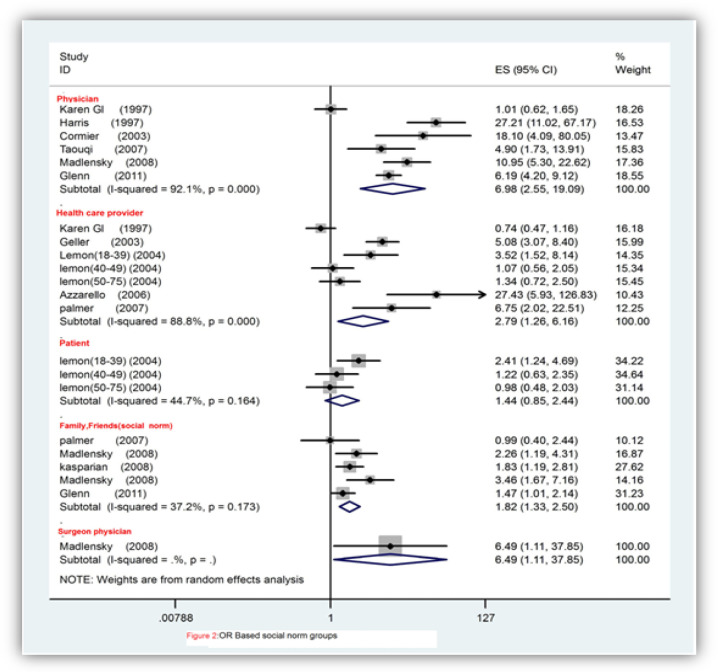
Forest Plot Illustrating Weighted Odds Ratio (OR) Using Random Effect Model for Intention to Screening or Screening Arranged by Groups' Subjective Norms

 Furthermore, there was a heterogeneity evidence which is shown in Figure 2 (Cochrane Q test, Q statistic = 62.91, P<0.00, I^2^=92.1). We carried out a subgroup analysis based on cutoff points for the cancer types and a study design analysis in order to find the source of heterogeneity. The Meta-analysis according to the OR showed that the PR is significantly related to screening based on the CRC (OR=6.07, 95% CI; 1.23–30.06; P=0.027) and prostate cancer (OR, 8.24, 95% CI; 3.25–20.90; P<0.001). There was an evidence of heterogeneity between studies in CRC (Cochrane Q test, Q statistic=54.53, P<0.001, I^2^=94.5) and prostate cancer (Cochrane Q test, Q test=1.87, p=0.171, I^2^=46.6). Furthermore, there was not enough information to reject the null hypothesis about between-study heterogeneity.

**Figure 3:**
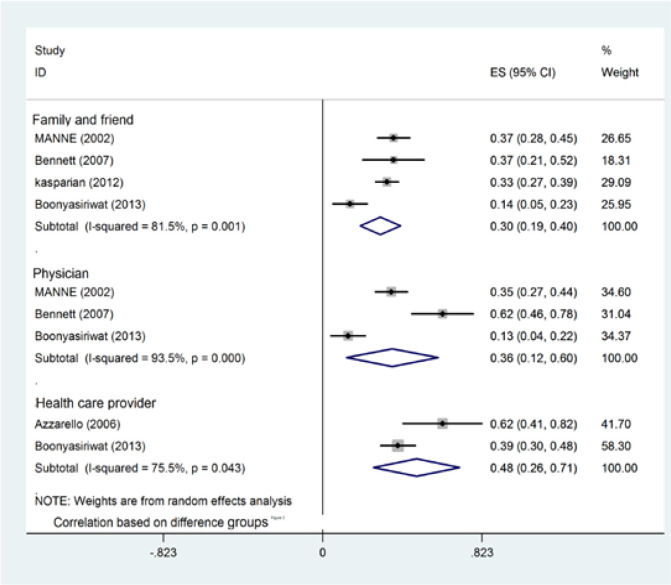
Forest Plot Illustrating Weighted Correlation (r) Using Random Effect Model for Intention to Screening or Screening Arranged by Groups' Subjective Norms

According to the meta-analysis, the correlation showed that the PR is significantly related to screening and increases preventive measures (r=0.36, 95% CI; 0.12-0.45, P<0.003). It was proven that there is a heterogeneity between studies in Q statistic as illustrated in Figure 3 (Cochrane Q test, Q statistic=30.63, P<0.001, I^2^=93.50). A subgroup and a study design analysis were performed to find the source of heterogeneity. The results of meta-analysis and the correlation based on cancers showed that Physician recommendation is significantly related to CRC (r=0.24, 95%CI; 0.02-0.46, P=0.03) and Breast cancer (r=0.62, 95%CI; 0.46-0.78, P<0.001). Heterogeneity between studies was investigated using Q statistic in CRC (Cochrane Q test, Q statistic=12.81, P<0.001, I^2^=92.2) and Breast cancer (Cochrane Q test, Q statistic=0, p=0, I^2^=0). Therefore, the null hypothesis about between-study heterogeneity was not rejected (Table 2).

*Health Care Provider (HCP) and Compliance with Screening*

**Figure 4:**
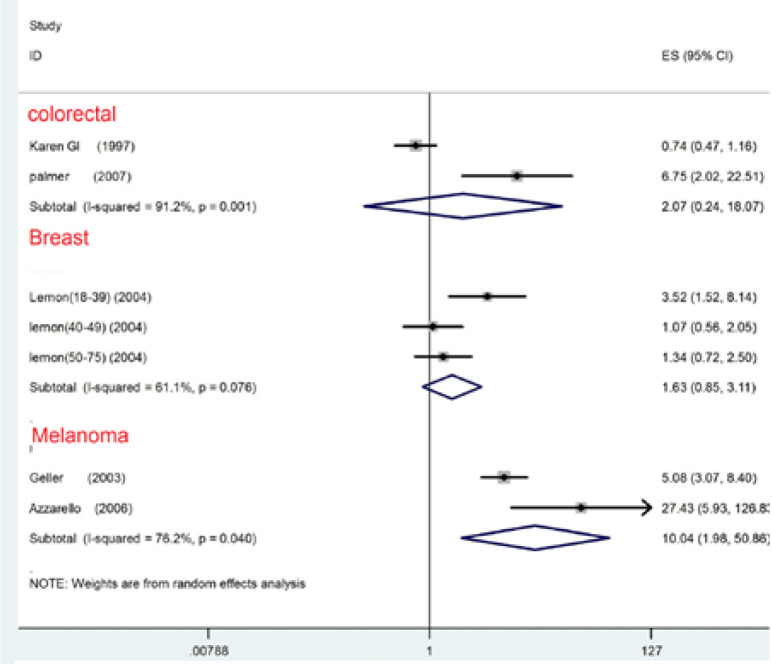
Forest Plot Illustrating Weighted Correlation (r) Using Random Effect Model for Intention to Screening or Screening Arranged by Type of Cancer

In some studies, HCP recommendation for screening compliance was assessed. For example, in Glanz study both the PR and the HCP were investigated (Glanz et al., 1999). In some other studies, the HCP as a physician or a nurse or as an assistant was identified (Azzarello and Jacobsen, 2007). In the study of Glanz, the HCP recommendation did not predict the intention to screening but in other studies it was predicted in the FDRs. The meta-analysis based on the OR showed that the HCP recommendation significantly increases the possibility of screening which is shown in Figure 2 (OR=2.79, 95% CI; 1.26-6.16; P=0.011). An evidence of heterogeneity between studies was found (Cochrane Q test, Q statistic =53.57, p<0.000, I^2^=88.8). In order to find the source of heterogeneity, a subgroup and a study design analysis were conducted. Based on the meta-analysis and the OR, it was found that the HCP recommendation does not show a significant increase in the possibility of screening in the CRC (OR =2.07, 95% CI; 0.24-18.01, P=0.509) (Figure 4) and Breast cancer (OR=1.63,95% CI;0.85-3.11, P=0.13); however, it significantly increases the possibility of screening in Melanoma (OR =10.04,95% CI;1.98-50.86, P=0.005). Heterogeneity was found to be existed among 3 types of cancers; CRC (Cochrane Q test, Q statistic =11.37, p<0.001, I^2^=91.2), Breast (Cochrane Q test, Q statistic=53.57, p=0.076, I^2^=61.1) and Melanoma (Cochrane Q test, Q statistic=4.21, p=0.040, I^2^=76.2). More information was needed to reject the null hypothesis of between-study heterogeneity. 

The results of meta-analysis correlation indicated that HCP recommendation significantly increases the possibility of screening (r =0.48, 95% CI; 0.26-0.71, P<0.001). Heterogeneity was found which is shown in Figure 2 (Cochrane Q test, Q statistic =0.040=4.08, p=0.043, I^2^=75.5). The null hypothesis could not be rejected of between-study heterogeneity due to insufficient information.

*Family and Friends Recommendation and Compliance with Screening*

Family and friends are one of social groups that motivate the FDRs to undergo screening. These groups were analyzed in some studies (Glenn et al,2012; Kasparian et al., 2012; Manne et al., 2003; Palmer et al., 2007).Therefore, meta-analysis was carried out on social groups’ recommendation for screening. Forest plot showed the OR of social groups that significantly increases the possibility of screening by the FDRs as demonstrated Figure 2 (OR=1.82, 95% CI; 1.33–2.50, P <0.001). There was no heterogeneity evidence (Cochrane Q test, Q statistic =6.37, p=0.173, I^2^=37.2).

The correlation results showed that there was a direct relationship between groups and compliance with screening and a direct relationship between recommendation and compliance with screening (r=0.30.95%, CI019-0.40, P<0.001). There was a heterogeneity evidence (Cochrane Q test, Q statistic =16.22, p<0.001, I^2^=81.5).

Subgroup and study design analyses were performed based on cutoff points of cancer types in different studies in order to find the source of heterogeneity. There was a relationship between subjective norms (friend and family) recommendation and compliance with screening based on CRC (r=0.25, 95% CI=0.03-0.47, P=0.024), Breast cancer (r=0.37CI=0.21-0.52, P<0.001), and Melanoma (r=0.33 CI=0.19-0.40, P<0.001). Q statistic results revealed a heterogeneity between studies based on cancers: CRC (Q test=12.94, I2=92.3, p=0.000), Breast (Q test=0, I^2^=0, p=0) and Melanoma (Q test=0, I^2^=0, p=0). Lack of enough information did not allow the authors to reject the null hypothesis of between-study heterogeneity (Figure 4).

*Publication Bias and Sensitivity Analysis*

**Figure 5:**
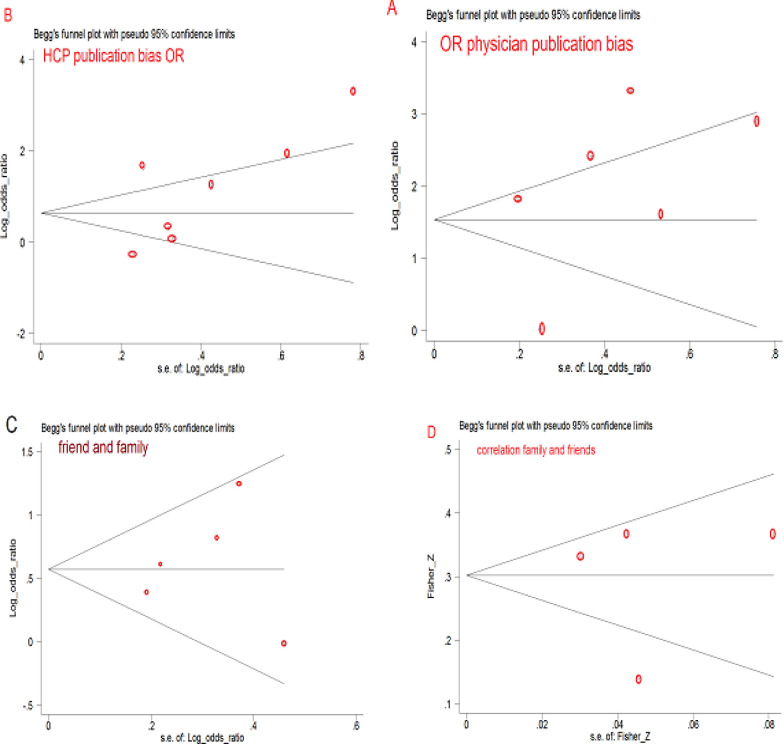
Beggs' Funnel Plots (with Pseudo 95%CI) of the Odds Ratio (OR) versus the SEs (Standard Errors) of the ORs that Evaluated the Relationship between Recommendation of Groups and Intention to Screening (or Screening). A, physician; B, HCP; C, Family and friends; D, Beggs’ funnel plots of Fishers Z versus the SEs (standard errors) of the Fishers Z that evaluated the relationship between recommendation of family and friends and intention to screening (or screening)

There was no publication bias evidence as indicated by the funnel plots (Figures 5(a) and (b)) and asymmetry tests for both the PR (Egger’s test: P=0·401 and Beggs test P=1) and the HCP recommendation (Begg’s test: P=0·230 and Eggers test: p=0.169). Therefore, publication bias was determined by the funnel test (Figure 5 and 4(c)) and asymmetry tests for subjective norms (family and friends) (Egger’s test: P=0·667 and Beggs test P=0.806). Furthermore, publication bias was ascertained by a correlation test in the PR (Beggs test =1 and Eggers test=0.832) and a correlation test in subjective norms (Beggs test =0.734 and Eggers test=0.892).

This may be due to the large population size or the limited number of studies included for meta-analysis. Since, there is a limited number of studies with meta-analysis on patients’ friend, family and surgeon, the publication bias or sensitivity analysis was not performed for these groups.

Begg’s funnel plots (with pseudo 95% CIs) of SEs (standard errors), OR and correlation evaluated the relationship between groups and screening intention.

**Table 1:** Observational Prospective Studies Eligible to Include in the Systematic Review and Meta-Analysis

Author-year	design	gender	Type of cancer	age	Sample size	Definition of Subjective norm	Main Result
Bennett 2007(26)	Cohort	FM	Breast	43.3	128	family and physician Recommendation (PR) to screening	REC Family (0R=0.37(0.27-0.52}) and physician (0R=0.62(0.46-0.78)) predicted intention to screening.
Glenn 2012(32)	Telephone surveys	Male	prostate	40-78	806	Physician and family Recommendation	PR (0R=1.82(1.44-2.21)) predicted intention to screening. Family (0R=1.82(1.44-2.21)) predicted intention to screening.
Boonyasiriwat 2013 (20)	survey	F/M	CRC	32–74	481	PR to colonoscopy	PR (0R=.14(0.05-.23)) predicted intention to screening.
K. C. 2000-(30)	Cross sectional	FM	Breast	28-78	110	Social influences to self-examination	social influence and thought are considered as barriers to intention of screening, however, it was not investigated in this article
Manne-2002(34)	Cross sectional	F/M	CRC	30-70	556	Family and physician encouragement screening	Family support predicted intention to screening (0R=.37(0.28-.45)) physician support predicted intention to screening (0R=.35(0.27-.44)
Geller-2003(39)	Cross sectional	F/M	melanoma	18-50	249	Health care provider (HCP) recommendation to self-examination	HCP recommendation predicted self-examination (0R=1.63 (1.12-2.13))
Azzarello-2006(31)	Cross sectional	F/M	melanoma	23-87	95	HCP recommendation to total cutaneous examination	HCP recommendation predicted cutaneous examination (0R=1.41 (0.22-2.63))
Cormier-2003(19)	Cross sectional	male	prostate	53 ± 9	138	PR to screening	PR (0R=1.81(0.89-2.72)) predicted intention to screening.
Glanz,1997(23)	survey	F/M	CRC	20-59	390	PR and HCP recommendation	PR (0R=-0.01(-0.48-0.50)) not predicted intention and HCP (0R=-0.30(-0.76-0.15)) not predicted intention to screening.
Hevey-2008(22)	survey	male	prostate	40-70	223	Hierarchal regression predicting intention to take PSA test if offered by the Physician	sr2 (%)=2 subjective norm not predicted intention to attend PSA screening
Kasparian-2008(33)	web-based survey	F/M	melanoma	16-80	1094	Social norm (family and friends motivate skin self-examination)	Social norm predicted skin self-examination (OR=1.83 (1.19-2.80))
Lemon-2004(27)	cohort	female	Breast	18-75	577	Patient and HCP recommendation	REC patient predicted mammography (0R=1.44 (0.85-1.44))and REC HCP predicted mammography (40-49years) (0R=3.52 (1.52-8.14))
Taouqi-2007(40)	cross sectional	F/M	CRC	30-70	138	Screening advised by the physician	PR ((0R=4.90(1.73-13.9)) predicted to screening
Harris-1997(41)	cross sectional	F/M	colon	30-90	225	HCP REC	REC HCP predicted intention to screening (0R=3.30 (2.40-4.21))
Palmer-2007(35)	Cross sectional	F/M	CRC	35-50	174	Medical recommendation Social norm	Health provider predicted to screening (0R=6.75 (1.89-21.02)) Social norm predicted to screening (0R=0.99(0.41-2.49))
Bronner-2013(29)	cross-sectional study	F/M			318	Physician and family REC Friends ,Colleagues and Neighbors	All screeners were more likely to be offered a medical recommendation (N = 87, 63.5%, P < 0.0001), to be encouraged by family (N = 75, 54.7%) than non-screeners (N = 56, 30.9%; N = 72, 39.8%; N = 128, 70.7%, respectively). no influence was observed for reference social groups such as friends, colleagues, neighbors, and religious or spiritual mentors, however, it was not investigated in this article
Madlensky-2008(21)	Interview telephone	F/M	CRC	34-80	368	PR, social group and family recommendation	PR (0R=10.95 (5.30-22.62)), social group (friend, coworker, neighbors). (0R=2.26 (1.19-4.31)}, family (0R=3.46 (1.67-7.16)) predicted screening.
Garcia 2011(28)	Cross sectional	F/M	CRC	40-60	334	PR	Association to visit physician and trend to participation screening not statistically significant, but did not have data.

**Table 2:** Study Quality and Risk of Bias Assessment Using the Modified Newcastle-Ottawa Scale (25).

	selection					Outcome			
Author (year)	Representativeness of sample	sample	Nonrespondent	Ascertainment of exposure	Comparability	Assessment of the outcome	Statistical test	score	Quality
Bennett 2007 (26)	*	*	*	*	*	*	*	7	High
Glenn 2012 (32)	*	*	*	*	*	**	*	8	High
Boonyasiriwat 2013 (20)	*	*	-	*	**	*	*	7	High
Manne-2002 (34)	*	*	*	**	*	**	*	9	High
Geller-2003 (39)	*	*	*	-	**	*	*	7	High
Azzarello-2006 (31)	*	*	*	**	**	*	*	9	High
Cormier-2003 (19)	*	*	*	*	**	*	*	8	High
Glanz,1997 (23)	*	*	*	*	**	*	*	8	High
Kasparian-2008 (33))	*	*	*	**	*	**	*	9	High
Lemon-2004 (27)	*	*	*	*	**	**	*	9	High
Taouqi-2007 (40)	*	*	-	**	**	**	*	9	High
Harris-1997 (41)	*	*	*	*	**	*	*	8	High
Palmer-2007 (35)	**	*	*	*	**	*	*	9	High
Madlensky-2008 (21)	*	*	*	*	**	*	*	8	High

## Discussion

The findings showed that subjective norms significantly increased the presence of cancer patients’ FDRs for screening. Furthermore, a significant linear association was shown between subjective norms and attendance in screening.

The prediction of screening attendance in people in a particular high-risk condition was mentioned in the literature review (How well do the theory of reasoned action and theory of planned behavior predict intentions and attendance at screening programs? A meta-analysis).

Therefore, the literature review of meta-analysis assessed subjective norms for understanding health behaviors.

Cooke et al., (2008) found subjective norms which had medium-sized relationships with the intention. McEachan et al., (2016) indicated that subjective norms were significant independent predictors of behavior in regressions and all preventive behaviors. Even in an intervention study, using subjective norms along with other factors is proposed to increase the likelihood of screening (Kiviniemi et al., 2011). The results showed that social groups and physicians enhance the screening attendance (OR, 2.79 CI; 1.26–6.16; P =0.011). In Beydouns’ study, which is a systematic review, the physician recommendation predicted colorectal cancer screening. In other studies, the physician recommendation is considered as one of the predictors of screening, which is in line with the results of this study. Furthermore, the heath care provider was one of the effective groups for encouraging the FDRs to screen in this study and there was a direct and significant relationship between the heath care provider recommendation and screening. The results indicated the importance of the heath care provider, and in a number of studies a doctor has also been referred to as a heath care provider. However, in most studies, nurses or a member of the health care teams is the heath care provider. The heath care provider plays an important role for the patients and relatives due to providing helpful information (Cooke and French, 2008; McEachan et al., 2016). The lack of physician recommendation for screening is a barrier to the presence of at-risk people. Furthermore, physicians do not know the level of participation of cancer patients’ relatives in screening (Geller et al., 2003; Kiviniemi et al., 2011). Further education can solve this problem (Harris and Byles, 1997; Taouqi et al., 2010). Friends and relatives’ recommendations can also encourage screening. Social pressure involves doctors, caregivers and relatives, which can greatly affect screening for relatives.

Research studies conducted on cancer screening were included in this study (breast, colorectal, prostate and melanoma). Cooke et al., (2008) included studies on some other types of cancers (breast, cervical, colon). One of the strengths of this study was the differentiation of the effectiveness of groups such as physicians, family and patients, which were systematically studied. In other studies, the subjective norm is not evaluated for certain groups. For example, the influence of doctors, friends and relatives is generally considered a subjective norm. However, the total number of these groups and the motivation to comply are regarded as subjective norms.

In this study, the impact of social pressure on cancer patients was investigated in particular, which was not observed in other studies.

Heterogeneity was significantly observed between studies, only a few of which were described by mediators. Examining different types of cancers and combining different studies can lead to heterogeneity as well as using various methods for measuring social pressure. In some studies, a social norm construct was investigated and in some others, the recommendations of influential people were examined; also, the behavioral outcomes were different. In addition, different structures of the questionnaires and different evaluation of subjective norm can affect heterogeneity. Recent attempts to develop standard measurement tools are also addressed, which is absent in other review studies. Regardless of the full definition of the subjective norm, it can also lead to the heterogeneity between studies. 

## Study Limitations

One of the limitations of this study was the lack of using the same subjective norms and investigating them in a variety of ways and with different questions.

## Clinical Implications

Eventually, this study showed that a subjective norm can be used as a method of encouraging FDRs to be screened along with other methods. However, according to the review studies, it has a less central role than attitude and perceived behavioral control.

## List of Abbreviation

OR: Odds Ratio; HCP: Health Care Provider; CRC: Colorectal; FDR: First-Degree Relative; PRISMA: preferred items to report systematic reviews and meta-analyses; SE: standard error.
